# Supplementary figures and images for: Start up: A French program to support patients with pulmonary arterial hypertension during the adjustment of prostanoids to the individualized optimal dose
Source: PLoS One. 2025 Oct 29;20(10):e0331008. doi: 10.1371/journal.pone.0331008 (PMC12571280; doi:10.1371/journal.pone.0331008)

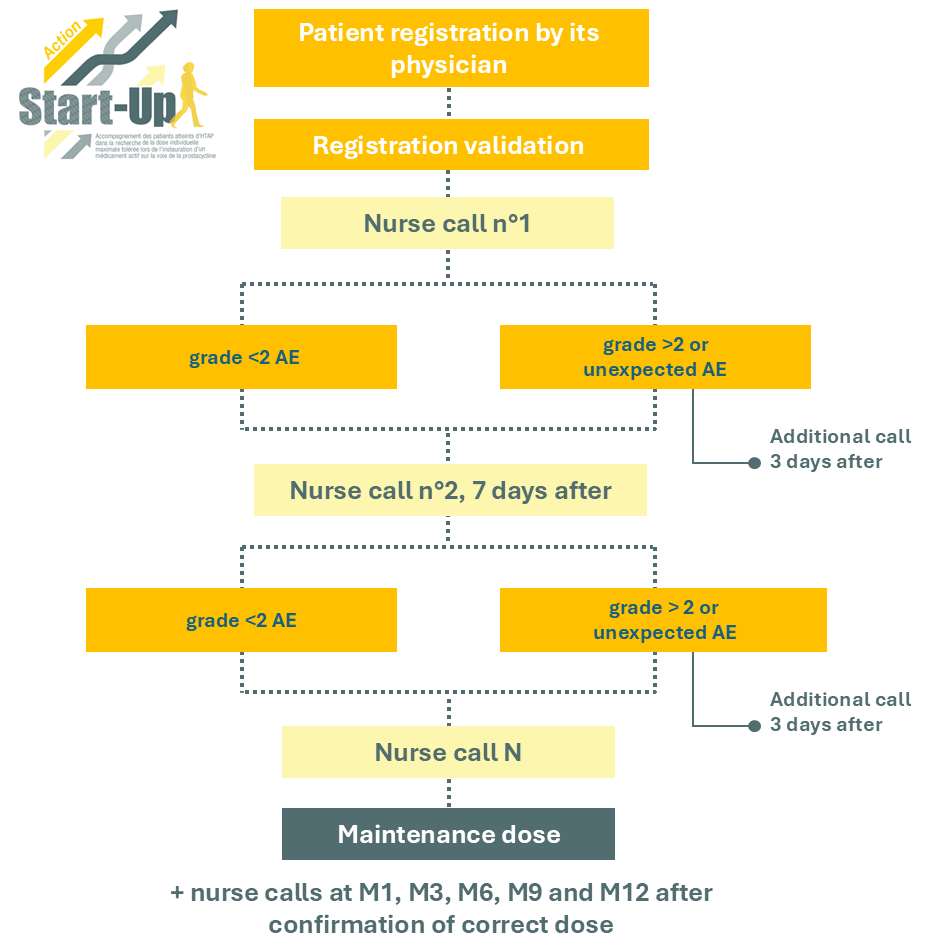

Supplement: S1 Fig — (TIF) [file pone.0331008.s001.tif]
