# Supplementary material for: Start up: A French program to support patients with pulmonary arterial hypertension during the adjustment of prostanoids to the individualized optimal dose
Source: PLoS One. 2025 Oct 29;20(10):e0331008. doi: 10.1371/journal.pone.0331008 (PMC12571280; doi:10.1371/journal.pone.0331008)
Supplement: Attachment — Submitted filename: Response to reviewers_Vf.docx [file pone.0331008.s003.docx]

Response to reviewers

Dear PlosOne editor, dear reviewers,

We would like to thank you for these constructive comments on our work. Please find below our responses to all of your concerns.

**Academic Editor comments**:

Style requirements: Requirements regarding style and file naming are now met in the updated version.

Funding statement: All funding is declared in the funding statement. The statement “There was no additional external funding received for this study” has been added to the reviewed cover letter.

Data accessibility: Data are issued from the Start Up patient support program developed by Patientys. Patient gave their written consent for the use of their data, however they did not agree specifically for public availability of their unaggregated data. To meet PlosOne requirements, data can be available upon request at [privacy@patientys.com](mailto:privacy@patientys.com). The section has been updated in the submission form.

Ethics statement: Our ethics statement has been updated in the methods section with complete information about the institutional body.

Reference list: The reference list has been checked to ensure that it is complete and correct.

**Reviewer#1 comments**

Objective of the manuscript: *The Start Up program was first designed to bring support to patients and their medical teams while initiating prostacyclin. The objective of the current study was to report the existence of such patient support program and give real world data supporting the hypothesis that an external support is beneficial to patients and physicians in treatment titration. Our data shows that the external nurse allows for a personalized titration pattern following AE reported by patients which may participate to a higher rate of patient reaching their optimal dose and a lower discontinuation rate. In addition, the time spent by the external nurse is time saving for medical teams and highly appreciated.*

AE assessing: *During the phone calls, using guidelines determined by the scientific committee, the nurse drives the patient interview to identify and grade AEs. There is a specific focus on prostanoids associated AEs, in order to give insights to the medical team of the patient that will take decision regarding the treatment. The methods section has been detailed.*

Medications use: *Medications to treat AEs were only manage by the medical team of the patient, in regard to the AEs declared by the patient. Some medical teams anticipated and gave to their patient a prescription for medication to treat AEs while introducing Selexipag. The PAH nurse of the Start Up program may solicit the physician to send a prescription if needed but do not take any medical decisions. The methods section has been detailed.*

Discontinuation rates compared to current literature *In the Start Up program, 78 patients (19%) discontinued their treatment, before reaching their optimal treatment dose. Among them, 35 patients (9%) reported a treatment discontinuation due to adverse events during the titration phase.*

*In the Phase 3 clinical trial (GRIPHON), 14,3% of patient reported a treatment discontinuation related to adverse event. Among the several real-world data studies, more relatable to the Start Up program, the discontinuation rate due to adverse events varies from 20% in the EXPOSURE study and 22% in the SPHERE registry study up to 32% in the GRIPHON open label study. However, they monitor discontinuation after the titration period, making a comparison more difficult with our study. We could only speculate that because adverse events occur more frequently during the dose-adjustment phase (Sitbon et al., 2015), treatment discontinuation may have been higher during the titration phase.*

*Therefore, the discontinuation rate observed in the Start Up study may be lower than other real world data studies, supporting that the close patient monitoring may participate to less treatment discontinuation. 🡪 The discussion section has been detailed.*

Minor points:

- “Benefited” 🡪 “Received”: *modified*
- Avoid the use of “him” when referring to physician: *modified*
- Line 164 and 166: Add “S” to patient: *modified*
- Line 267: ass “of”: *modified*

Patients’ satisfaction completion: *The satisfaction questionnaire was only implemented later in 2018, as described in the Methods section and sent to 249 patients out of the 302 patients that reached their optimal dose. In addition, patients must fill-out the satisfaction questionnaire and send it using a prepaid envelope to the PAH nurse. This postal sent by the patient may explain the low completion rate.*

Completion rate of health care providers: *Unfortunately, amongst healthcare providers, the distribution of the questionnaires was not controlled. While the questionnaire was sent by email to 64 physicians, we received answers from nurses and pharmacists suggesting email forwarding. We cannot therefore provide a response rate among healthcare professionals.*

**Reviewer#2 comments**

Page 2, line 27, write out the CTCAE v4.0. 🡪 *detailed*
Page 5, line 71, write out HTaPFrance. 🡪 *detailed*
Page 5, the timeline described in line 76-81 could be more easily described if Figure 1 is referenced. I may read badly, but the numbers do not fit with the description in results from line 146 onwards. 🡪 *Line 76-81 aimed to describe the 2 populations of patients that benefited or not of the additional follow-up of 1 year. Line 149 and followings describe the whole population and detail the patients that did not reached their individualized dose. For a better clarity the Methods section is detailed and figure 1 referenced.*
Page 8, line 143, what is MR004 reference methodology (no reference given). 🡪 *detailed*
Page 12, line 184-186, I miss the definition of high, medium, low in the Methods section. Were these cut-offs decided a priori? 🡪 *This is aligned to the GRIPHON study, Phase III trial for Selexipag. The Methods section has been detailed.*
Page 16, “alert from the French drug regulatory agency”, please explain (not only in the footnote to Figure 1). 🡪 *Explained in the Results section*
Page 16, line 238, very few patients answered the rating, please discuss if the findings are representative with such a low response rate. Same page, what was the response rate amongst health professionals? LATER, I see the discussion under limitations on page 21. But the authors could speculate more any effect of biases. 🡪 *Discussion more detailed. The low response rate among patients may be due to the data collection method for satisfaction. Patients had to return the questionnaire in a pre-stamped envelope. Unfortunately, amongst healthcare providers, the distribution of the questionnaires was not controlled. While the questionnaire was sent by email to 64 physicians, we received answers from nurses and pharmacists suggesting email forwarding. We cannot therefore provide a response rate among healthcare professionals.*

Figure 2, please give an explanatory footnote. As now, the figure is not understandable. 🡪 *Detailed*

Figure 4, what number is correct? In text, out of 302 reaching dose, 86 answered. In Figure 4, 249 questionnaires were sent. Please check and be careful with numbers and be consistent! 🡪 *Of the 302 patients that reached their optimal dose, the questionnaire was sent to 249 patients. This is due to the implementation of the satisfaction questionnaire later in the program resulting in early participants not filling the questionnaire. The results section has been detailed to be consistent.*
